# Spasmolytic Activity of Fruits of *Tamarindus indica* L

**DOI:** 10.4103/0975-1483.66805

**Published:** 2010

**Authors:** N Ali, SWA Shah

**Affiliations:** Department of Pharmacy, University of Malakand, Chakdara, Dir (Lower). Khyber Pakhtunkhwa, Pakistan

**Keywords:** Calcium channel blocking, EC_50_ values, spasmolytic, *Tamarindus indica*

## Abstract

**Objective:**

To study the possible effects of methanolic extract of fruits of *Tamarindus indica* on rabbit’s jejunum preparations.

**Materials and Methods:**

The study was carried out on rabbit’s jejunum preparations. Methanolic extract of fruits of *T. indica* was applied in different doses. Potassium chloride (KCl 80 mM)-induced contractions were relaxed by the extract. For determination of possible mode of action of relaxing effects, calcium chloride curves were constructed in decalcified tissues using K^+^ normal and followed by K^+^ rich solution. The curves were then compared with verapamil.

**Results:**

Relaxant effects on KCl-induced contractions were prominent at a concentration of 5.0-10.0 mg/ml (*P*<0.05). A right shift at a concentration of 3.0 mg/ml (EC_50_ ± SEM = 1.98 ± 0.03) and 10.0 mg/ml (EC_50_ ± SEM = 1.79 ± 0.05) versus control (EC_50_ ± SEM = 2.33 ± 0.058) resembled the effects of verapamil on the calcium chloride curves.

**Conclusion:**

The results confirmed its possible mode of relaxing effects i.e. through calcium channel blockade.

## INTRODUCTION

*Tamarindus indica* is a plant of great medicinal importance and is used in the treatment of bacterial infections, gastrointestinal disorders, jaundice, and fever.[1–5] Other reported pharmacological activities are antifungal,[6] antiinflammatory, and antidiabetic activities.[7] Recently, pod shells of *T. indica* are tried for the purification of industrial waste water from heavy metals like chromium.[8]

The plant materials especially the stem bark decoction is used as gargle for sore throat. Decoction of leaves of *T. indica* along with the aerial parts of *Stercufta africana* is used in the management of diarrhea.[9] Cholesterol-lowering effects of the fruits of the plant has also been documented.[10]

The fruits and seeds of the plant are used as food stuff and it has been suggested that the plant is a rich source of vitamin B complexes, carbohydrates, and proteins.[11] Reported compounds from *T. indica* are lupeol, lupanone,[12] and orientin.[13] Since its fruits are used in different food items like chutneys, achars, jams, and candies, hence, we carried out our current work for possible effects on rabbit’s jejunum preparations to explore its folkloric uses on scientific grounds.

## MATERIALS AND METHODS

### Collection and extraction of plant materials

The dried ripe fruits of *T. indica* were purchased from the local market. The plant was identified by Professor Dr Jehandar Shah, vice chancellor Shaheed Benazir Bhutto University, Sheringal Dir Upper. A voucher specimen (TI-01-2009) has been deposited to the herbarium of University of Malakand. The plant materials were washed with distilled water. After drying, the fruits were chopped into small pieces so that its seeds remain intact and were soaked in commercial grade methanol. After 15 days, the materials were filtered through ordinary filter paper. The process was repeated thrice. Combined the filtrates and concentrated to brownish crude extract at 40 °C using a rotary evaporator.

### Drugs

Analytical grade chemicals were used in the experiments. Drugs used in the experiments were dissolved in distilled water and all solutions were freshly prepared on the same day of experiments. Acetylcholine was purchased from BDH Chemicals, Poole, England. Rests of the chemicals were purchased from E. Merck Germany.

### Animals

Local breed rabbits of either sex were used in experiments and their weights were in range 1.0-1.4 kg. The animals were housed at the “Animal House of University of Malakand” complying with standards mentioned in the “Animals Bye-Laws 2008 of the University of Malakand (Scientific Procedures Issue- 1).” Standard diet and tap water was given to the animals. The animals were kept without food, 24 h prior to start of the experiments and they were given only water.

### Data recording

Data related to intestinal responses were recorded using Force Transducer (Model No: MLT 0210/A Pan Lab S.I.) attached with Power lab (Model No: 4/25 T) AD Instruments, Australia. Data were recorded at range of 20 mv, low pass 5 Hz × 10 gain using input 1, rate 40/S.

### Statistical analysis and interpretation of data

Chart 5 for Windows supplied with Power lab purchased from AD Instruments, Australia, was used for the interpretation of graph tracings. Microsoft-XL Sheet was used to calculate mean and standard error mean (SEM) of the data (not shown). Student’s ‘t’ test was used to compare the data, and ‘P’ value less or equal to 0.05 was considered as statistically significant.

### Rabbit’s jejunum preparations

Experiments on rabbit’s jejunum preparations were carried out in following fashion, as per our previous work done.[14] The animals were slaughtered and their abdomen was opened and portion(s) of jejunum were isolated and kept in tyrode’s solution. The solution was aerated with carbogen gas (5% Carbon dioxide and Oxygen mixture)[15] to keep the tissues live and ready for use. Preparations of about 1.5 cm length were mounted in 10 ml tissue bath containing tyrode’s solution and were stabilized for about 25-30 min. All the protocols were carried at 37 ± 1°C and aerated with the carbogen gas. A pressure of 1 g was applied to the tissues. When a reproducible response was observed, then we tried the crude methanolic extract at dose(s) of 0.01, 0.03, 0.1, 0.3, 1, 5 and 10 mg/ml (final bath solution). Constituents with their respective concentrations (mM) of the tyrode’s solution were KCl 2.68, NaCl 136.9, MgCl_2_ 1.05, NaHCO_3_ 11.90, NaH_2_PO_4_ 0.42, CaCl_2_ 1.8, and glucose 5.55. Quiescent sub-maximal doses of acetylcholine (0.3 μ M) to the tissues were used whence needed for keeping the tissue viable and live.

### Spasmolytic and calcium channel blocking activity

As the extract produced a dose-dependent fall in spontaneous activity, hence, we tried to explain the possible mode of action. The tissues were pre-treated with a high concentration of KCl (80 mM in final bath volume) to depolarize and get them in position of sustained contractions.[16] Then we applied the extract on the tissues, in a cumulative manner, to obtain a dose response curve and their relaxation was expressed as % of the KCl-induced contractions.[17]

A partial relaxation was observed that was further confirmed by performing another series of experiments in calcium free tyrode’s solution having EDTA (0.1 mM). The tissues were then exposed to potassium rich tyrode’s solution for 30 min having following composition (mM): KCl 50, NaCl 91.04, MgCl_2_ 1.05, NaHCO_3_ 11.90, NaH_2_PO_4_ 0.42, glucose 5.55, and EDTA 0.1. Earlier, all the tissues were stabilized in normal tyrode’s solution for at least 30 min. We constructed controlled calcium response curves in the decalcified tissues with cumulative addition of Ca++ in a concentration range 1 × 10-4 to 256 × 10-4 M. The decalcified tissues were then exposed to the extracts at least for period of 1 h to get the calcium concentration response curve. The extract treated calcium curves were then plotted versus controlled calcium curves. In other series of experiments, the decalcified tissues were exposed to different concentrations of verapamil (3 × 10-9 to 1 × 10-6M) in cumulative fashion versus control curves in the decalcified tissues. The curves were then compared with that of verapamil for confirmation of possible calcium channel blocking activities.

## RESULTS AND DISCUSSION

**Figure 1:**
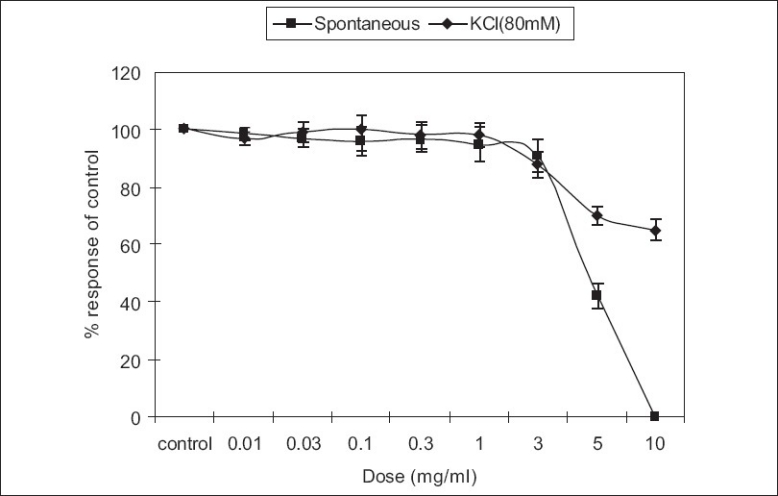
Dose response curves of crude extract of *Tamarindus indica* on spontaneous contractions of isolated rabbit’s jejunum preparations. Extract effects on KCl-induced contractions are also shown. All values are mean ± SEM (n=6).

**Figure 2:**
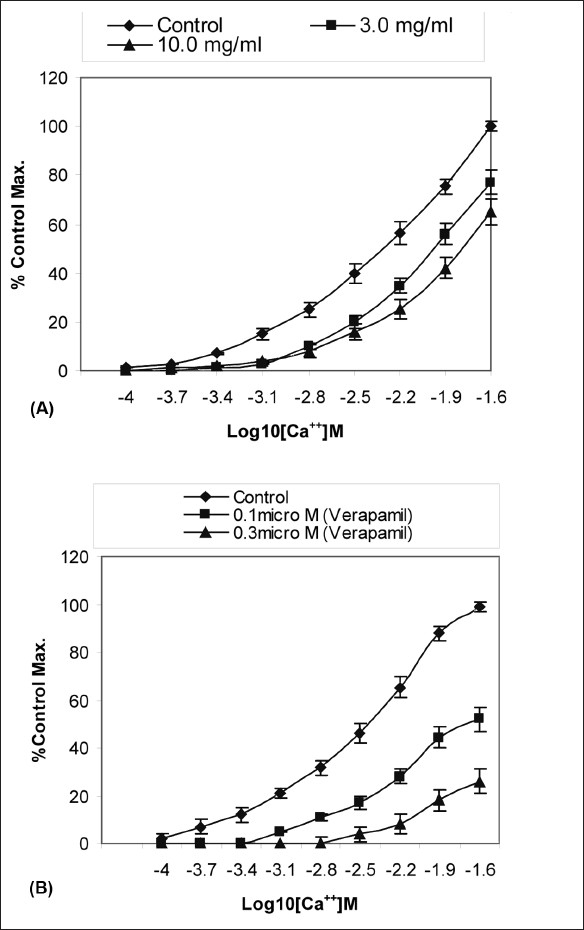
Dose response curves of Ca++ in the absence and presence of increasing doses of (A) extract of *Tamarindus indica* and (B) verapamil in isolated rabbit’s jejunum preparations. All values are mean ± SEM (n=6).

The effects of different concentrations of extract of *T. indica* are shown in Figure 1. The extract produced dose-dependent relaxing effects on spontaneous rabbit’s jejunum preparations. Prominent relaxations in the spontaneous responses were observed at a concentration of 5.0 mg/ml (EC_50_ ± SEM = 4.6 ± 0.37). However, at a concentration of 10.0 mg/ml, the extract produced complete relaxation of spontaneous response(s). To explain its possible mode of action, sustained contractions in the tissues were produced by KCl (80 mM). The contractions were relaxed by the extract in similar fashion. The extract at a concentration of 10 mg/ml relaxed the contraction upto 65% of the control [Figure 1]. The contractile effects of the smooth muscles of the intestines are due to the cytosolic free calcium levels and there is an exchange of calcium between extra-cellular and intra-cellular calcium stores. The voltage-dependent calcium channels (VDCs) are responsible for the influx of calcium into the sarcoplasmic reticulum.[18–21] This leads to periodic depolarization and repolarization of the intestinal tissues that account for its spontaneous responses. Hence, we tried to explain the mode of the relaxation possibly by calcium channel blockade.[22] As a positive relaxing effect on the KCl-induced contractions does not always imply for calcium channel blockade,[23] hence, the results were confirmed by plotting the calcium chloride curves versus in decalcified tissues using verapamil as standard. The results are summarized in Figure 2. According to Figure 2A, it is evident that the relaxing effect of extract at 3.0 mg/ml was 77% of the control maximum. The effect of extract was 65% of control maximum at 10.0 mg/ml. It is noteworthy that the effects of tested doses were suprimposible (almost same) at a calcium concentration 16 × 10^−4^ M i.e. (Log10 [Ca^++^] M= −2.8). At concentration log10 [Ca^++^] M= −2.2 and onward, a prominent right shift was observed. According to Figure 2A, a right shift at a concentration of 3.0 mg/ml (EC_50_ ± SEM = 1.98 ± 0.03) and 10.0 mg/ml (EC_50_ ± SEM = 1.79 ± 0.05) versus control (EC_50_ ± SEM = 2.33 ± 0.058) resembles like that of the curves shift of verapamil, a standard calcium channel blocker, at a concentration of 0.1 μ M and 0.3 μ M [Figure 2B].[24] This right shift of the extract at 3.0 and 10.0 mg/ml confirms the calcium channel blocking activity of *T. indica*.

## CONCLUSION

The results confirm the folkloric uses of *Tamarindus indica* in management of diarrhea.

## References

[1] Souza A, Aka KJ (2007). Spasmogenic effect of the aqueous extract of *Tamarindus indica* L.(caesalpiniaceae) on the contractile activity of guinea-pig *Taemia coli*. Afr J Trad Cam.

[2] Ali MS, Ahmad VU, Azhar I, Usmanghani K (1998). Chemo-tropism and antimicrobial activity of *Tamarindus indica*. Fitoter.

[3] Ferrara L (2005). Antioxidant activity of *Tamarindus indica* L. Ingr Alimen.

[4] Kheraro J, Adam JG, Vigot et Frères (1974). La pharmacopée sénégalaise traditionnelle, plantes médicinales et Toxiques.

[5] Kobayashi A, Adenan MN, Kajiyama SI, Kanzaki H, Kawazu K (1996). A cytotoxic principle of *Tamarindus indica*, di-n-butyl malate and the structure-activity relationship of its analogues. J Biosci.

[6] Pousset JL, Ellipses (1989). Plantes médicinales africaine, Utilisations Pratiques.

[7] Khanzada SK, Shaikh W, Sofia S, Kazi TG, Usmanghani K, Amina A (2008). Chemical constituents of *Tamarindus indica* L.Medicinal plant in Sindh. Pak J Bot.

[8] Ahalya N, Kanamadi RD, Ramachandra TV (2008). Biosorption of Chromium (VI) by *Tamarindus indica* pod shells. J Environ Sci Res Int.

[9] Kokwaro O (1976). Medicinal Plants of East Africa, East African Literature Bureau, Kampala.

[10] Marufiftekhar AS, Rayhan I, Quadir MA, Akhteruzzaman SA, Hasant A (2006). Effect of *Tamarindus indica* fruits on blood pressure and lipid-profile in human model: An *in vivo* approach. Pak J Pharm Sci.

[11] Dalimartha S (2006). Atlas Tumbuhan Indonesia, Jilid 4.

[12] Iman S, Azhar I, Hasan MM, Ali MS, Ahwed SW (2007). Two triterpenes lupanone and lupeol isolated and identified from *Tamarindus indica* L. Pak J Pharm Sci.

[13] Koeppen BH, Roux DG (1965). C-Glycosylflavonoids.The chemistry of orientin and iso-orientin. Biochem J.

[14] Bashir A, Niaz A, Shumaila B, Sadiq A, Ibrar M, Jamshid K (2009). Cholinomimatic and calcium channel blocking activity of the aerial parts of *Tylophora hirsuta* wall. J Chem Soc Pak.

[15] Qayum A (2004). Isolated preparations. Guidelines and instructions. In: Fundamentals of Experimental Pharmacology.

[16] Farre AJ, Columbo M, Fort M, Gutierrez B (1991). Differential effects of various Ca++ antagonists. Gen Pharmacol.

[17] Van Rossum JM (1963). Cumulative dose-response curves II: Techniques for the making of dose-response curves in isolated organs and the evaluation of drug parameters. Arch Int Pharmacodyn Ther.

[18] Karaki H, Wiess G (1983). Mini-review: Calcium release in smooth muscles. Life Sci.

[19] Bolton TB (1979). Mechanism of action of transmitter and other substances on smooth muscles. Physiol Rev.

[20] Carl A, Lee HK, Sanders KM (1996). Regulation of ion channels in smooth muscles by calcium (Invited Review). Am J Physiol.

[21] Godfraind T, Miller R, Wibo M (1986). Calcium antagonism and calcium entry blockade. Pharmacol Rev.

[22] Gilani AH, Bukhari IA, Khan RA, Khan A, Ullah F, Ahmad VU (2005). cholinomimetic and calcium channel blocking activities of *Carthamus oxycantha*. Phytother Res.

[23] Kobayashi S, Kitazawa T, Somlyo AE, Somlyo AP (1989). Cytosolic heparin inhibits muscarinic and α-adrenergic Ca++ release in smooth muscle. J Biol Chem.

[24] Cortes AR, Delgadillo AJ, Hurtado M, Dominguez-Ramirez Am, Medina JR, Aoki K (2006). The antispasmodic activity of *Buddleja scordioides* and *Buddleja perfoliata* on isolated intestinal preparations. Biol Pharm Bull.

